# Short-term effect of different time interval between self-expanding metallic stent and surgery for left-sided malignant colorectal obstruction

**DOI:** 10.3389/fgstr.2022.1059916

**Published:** 2022-11-16

**Authors:** Jiawei Zhang, Mingli Su, Dezheng Lin, Qinghua Zhong, Jiancong Hu, Jiaxin Deng, Miwei Lv, Tian Xu, Juan Li, Xuefeng Guo

**Affiliations:** ^1^ Department of Endoscopic Surgery, The Sixth Affiliated Hospital, Sun Yat-sen University, Guangzhou, China; ^2^ Guangdong Provincial Key Laboratory of Colorectal and Pelvic Floor Diseases, The Sixth Affiliated Hospital, Sun Yat-sen University, Guangzhou, China; ^3^ The Medical College of Xizang Minzu University, Xianyang, Shaanxi, China

**Keywords:** neoadjuvant chemotherapy, bridge to surgery interval, time interval, left-sided malignant colorectal obstruction, self-expanding metallic stent

## Abstract

**Background:**

The optimal time interval between self-expanding metallic stent (SEMS) placement and surgery in patients with left-sided malignant colorectal obstruction (LMCO) remains controversial. Intestinal obstruction and SEMS placement would lead to intestinal edema, local tumor infiltration, and fibrosis, which may have a certain impact on elective surgery. Although prolong time interval would reduce relative complications, the risk of tumor progression must be taken into account. Therefore, our study proposes whether there is a difference in short-term postoperative complication outcomes between waiting for an interval of ≤4weeks compared with an extended interval for neoadjuvant chemotherapy followed by surgery.

**Methods:**

All patients who underwent SEMS placement as BTS treatment for LMCO between January 2012 and December 2021 were retrospectively identified. The primary outcomes of this study were short-term clinical postoperative complications (Clavien-Dindo grading ≥II).

**Results:**

Of the 148 patients, 70.27% of patients underwent surgery ≤4 weeks of SEMS placement (Group 1) while 29.73% of patients underwent surgery >4 weeks of SEMS placement (Group 2). After SEMS placement, the patients in Group 2 received neoadjuvant chemotherapy and then elective surgery. Significant differences were observed between both groups (Group 2 vs Group 1) for postoperative complications (Clavien-Dindo grading ≥II, 2.3% vs 14.4%, p=0.040), postoperative bowel function time (p<0.001), postoperative hospital stay (p=0.028) and total hospital stay (p=0.002).

**Conclusions:**

A bridging interval of >4 weeks between SEMS placement and surgery for LMCO has better short-term clinical outcome.

## Introduction

Colorectal cancer is one of the third most common types of cancer in the world and the second most deadly cancer (1, 2). It is reported that about 10-30% of colorectal cancer patients have intestinal obstruction and most of them occur in the elderly (3, 4). The most common site of colon cancer obstruction is the left side. Colorectal obstruction can cause intestinal edema and bacterial translocation, which could lead to metabolic disorders, malnutrition, anemia, acid-base imbalance, septicemia, peritonitis, intestinal necrosis and even perforation. It usually requires emergency surgery for urgent colonic decompression to relieve intestinal obstruction. However, emergency surgery is associated with higher morbidity and mortality than elective surgery (5, 6). Therefore, the use of self-expanding metal stents (SEMS) as a bridge to elective surgery (BTS) as an alternative to emergency surgery has received increasing attention in patients with left-side malignant colorectal obstruction (LMCO). The expected benefit of SEMS as a BTS is an opportunity to optimize patients’ preoperative clinical conditions, reduce postoperative complications, and increase the rate of primary anastomosis (7). After relief of the obstruction according to SEMS insertion, time is created for there to be an improvement in the patient’s nutritional status, and also for accurate preoperative staging. Besides, neoadjuvant chemotherapy is feasible according to individual differences of patients to reduce preoperative clinical stage. It can greatly enhance the ability to withstand surgical shock and reduce the risk of perioperative mortality. Therefore, the BTS strategy is now recognized as a safe and effective alternative for decompression of LMCO (8). However, the optimal time interval between SEMS placement and surgery is still controversial, and it is generally believed that it will take several weeks for patients to improve their clinical and intestinal conditions (9). In general, a delayed interval between SEMS placement and elective surgery allows for better perioperative recovery and reduced postoperative complications, but it makes elective surgery more difficult because SEMS placement would result in more local tumor infiltration and fibrosis, and may increase the risk of tumor dissemination. The latest European Society of Gastrointestinal Endoscopy (ESGE) guidelines recommend that the interval between SEMS implantation and elective surgery is about 2 weeks, but this is based on low-quality evidence and expert opinion (10).

In addition, the NCCN guidelines for colon cancer recommend neoadjuvant chemotherapy for patients with large lymph nodes or clinical T4b disease and adjuvant chemotherapy for patients with high-risk stage II disease (11). Other studies have also shown that neoadjuvant chemotherapy is safe and effective for patients with locally advanced colorectal cancer (12). It has been reported that receiving neoadjuvant chemotherapy after SEMS placement not only can eliminate micrometastases and reduce the size of the primary tumor, but also largely could avoid the activation of early growth factors after surgical stimulation and reduce the risk of tumor cell shedding during operation, which so as to provide good conditions for follow-up surgical resection (13). However, compared with the SEMS implantation in patients with LMCO, it is not clear whether prolonging the interval of SEMS implantation for neoadjuvant chemotherapy will affect the perioperative recovery of patients who undergoing elective surgery and postoperative operation. Few studies have reported this problem.

Therefore, we conducted this study to determine the optimal time interval between SEMS implantation and elective surgery, and to study whether the extension of SEMS implantation interval for neoadjuvant chemotherapy will affect the perioperative recovery of patients undergoing elective surgery and postoperative surgery, besides, it also to evaluate the short-term clinical results for patients undergoing early (≤4 weeks) and delayed (>4 weeks) surgery.

## Materials and methods

### Patients

This study was a single-institution retrospective study of LMCO patients who underwent elective surgery after SEMS placement in the Sixth Affiliated Hospital of Sun Yat-sen University from January 2012 to December 2021. We retrospectively collected baseline demographics and preoperative information. This included the patient’s age, gender, American Society of Anaesthesia (ASA) classification, location of obstructive lesions, clinical cancer stage, presence of neoadjuvant chemotherapy, preoperative carcinoembryonic antigen (CEA), preoperative albumin and hemoglobin, as well as postoperative CEA. Intraoperative variables gathered and analyzed included operation type, operation time, intraoperative blood loss, recovery time of bowel function, postoperative hospital stay, total hospital stay, ICU hospital stay, perioperative complications, and histopathological findings, total number of lymph nodes collected and any nodal disease involvement as well as any vascular, lymphatic or perineural invasion, surgical approach (open versus laparoscopic) and whether a stoma was created. Outcomes of endoscopic stenting were analyzed for the presence of any significant post stent complications (perforation, migration, clinical failure). Time interval between date of SEME placement and date of elective surgery was collected and analyzed.

The study was approved by the institutional ethics board of the Sixth Affiliated Hospital, Sun Yat-sen University (NO. 2022ZSLYEC-429). We confirm that we have obtained ethical approval to conduct the study as well as permission from the dataset, and the study was conducted in accordance with the provisions of the Declaration of Helsinki (as revised in Fortaleza, Brazil, October 2013). The obtained data were only collected and analyzed; however, detailed information was not released in public, and information confidentiality regulations were strictly adhered to.

### Definitions

Colon cancer was regarded as a lesion confirmed with adenocarcinoma arising from the cecum to the rectosigmoid colon. Of these lesions, cancer arising from the mid-transverse colon to the rectosigmoid colon as left-sided colon cancer, including splenic flexure, descending colon, sigmoid colon. Left colonic obstruction is diagnosed by clinical symptoms (bloating, pain, and inability to pass stool or gas), clinical examination, endoscopy, abdominal plain radiography, and abdominal computed tomography (CT). Bridging time was defined as from the date of SEMS placement to the date of surgery. Technical success of SEMS placement was defined as successful deployment of the SEMS through the obstructing lesion, radiographically confirmed stent expansion and clear visualization of the fecal passage. Clinical success was defined as significant colonic decompression on abdominal radiograph or CT, resolution of obstructive symptoms, and absence of SEMS-related complications. The recovery time of bowel function is the time from surgery to the first release of gas or defecation. The postoperative hospital stay is the time from a resection of colorectal tumor to discharge from hospital. Operation time is determined by the colorectal surgeon based on the general condition of the individualized patient.

### Inclusion and exclusion criteria

Inclusion criteria included patients with colorectal obstruction due to left-sided malignant colorectal cancer and received SEMS placement as a bridge to curative intent surgery. Exclusion criteria were bowel ischemia, peritonitis, suspected or imminent perforation, contraindication to endoscopic therapy, obstruction caused by non-colonic malignancy or benign disease, history of colectomy, and SEMS implantation in other hospitals.

### Treatment

All patients underwent standard colectomy and regional lymphadenectomy. The surgical approach, mode of operation and scope of resection are determined by the surgeon according to the location of the tumor, the stage of the tumor and the general condition of the patient. Depending on the location of the obstructive disease and the presence of intestinal edema, left colectomy, anterior resection, low anterior resection, subtotal colectomy, abdominal perineal resection, and Hartmann surgery are performed by our experienced colorectal surgeons in a single center.

All SEMS placement was performed by experienced endoscopes using WallFlex colonic stents (Boston Science) or Evolution colonic stents (Cook Ireland Limited). The appropriate length of the selected SEMS should be sufficient to cover the entire stenosis, extending about 2 cm beyond the two narrow edges. The placement of SEMS includes interventional placement and endoscopic placement. All patients were given enemas for bowel preparation before SEMS placement. The colon proximal to the stenosis was evaluated by water-soluble contrast enema and the vital signs and clinical status of patients were monitored throughout the perioperative period. After the insertion of SEMS, the improvement of obstruction was monitored by abdominal symptoms and abdominal X-ray.

In this study, 44 patients received neoadjuvant chemotherapy, of which 33 patients received a median of 4.00 courses (IQR, 3.00-6.00 courses) of FOLFOX (oxaliplatin 85 mg/m^2^; folinic acid 400 mg/m^2^, followed by 5-FU, as a 400 mg/m^2^ intravenous bolus then a 2400 mg/m^2^ infusion over 46 h, days 1 and 2 of a 14-day cycle), 5 patients received a median of 8.00 courses (IQR, 5.00-12.00 courses) of FOLFOXIRI (oxaliplatin 85 mg/m^2^ dissolved in 500 ml of 5% glucose solution for intravenous infusion for 2 hours; irinotecan 150-165 mg/m^2^ dissolved in 250 ml of 0.9% sodium chloride for intravenous infusion for 90 minutes; followed by intravenous infusion inject folinic acid 400 mg/m^2^ for 2 h, on the first day; 5-FU 2800 mg/m^2^, continuous intravenous infusion over 48 h; once every 2 weeks), 6 patients received a median of 2.50 courses (IQR, 1.75-3.75 courses) of XELOX (intravenous infusion of oxaliplatin 130 mg/m^2^ on day 1, oral capecitabine tablets 1000 mg/m^2^ from day 1 to day 14; rest for 1 week, as a complete cycle, continuous 2 cycles).

### Study outcomes

The primary outcomes of this study were short-term clinical postoperative complications (Clavien-Dindo grading ≥II). Secondary outcomes included postoperative hospital stay, ICU treatment, and bowel function recovery time, operative time, intraoperative blood loss, total hospital stay, postoperative carcinoembryonic antigen, and surgical approach, number of lymph nodes examined, number of metastatic lymph nodes, vascular invasion, lymphatic invasion.

### Statistical analysis

Categorical data were evaluated using chi-square or Fisher’s exact test, while numerical data were evaluated using Student’s t-test or Mann-Whitney U test. Chi-square test and Fischer’s exact test were used for univariate analysis of categorical data between groups. Continuous variables expressed as median and interquartile range were compared with the Mann-Whitney U test. Numerical variables were dichotomized according to clinical importance or the median value of each variable as cutoff. For pairwise or multiple comparisons, bonferroni correction was used. Variables that had a P-value <0.05 in univariate analysis were subjected to multivariate logistic regression analysis. All p-values are two-sided and p<0.05 was considered statistically significant. Medians and interquartile range (IQR) were used to present data on patients’ duration of bridging stent.

## Results

### Baseline characteristics

In this study, a total of 158 patients who diagnosis with LMCO and underwent SEMS placement between January 2012 to December 2021 were enrolled. 2 patients with technical failure and 8 patients who had unresectable metastatic left colorectal obstruction and were unable to tolerate surgery due to their poor systemic condition were excluded.

After excluding 10 patients who did not meet the inclusion criteria, a total of 148 patients were enrolled. 104 patients underwent surgery after a bridging interval of ≤4 weeks (Group 1), and 44 patients >4 weeks (Group 2). The patients in Group 2 received neoadjuvant chemotherapy and then elective surgery after SEMS placement. The baseline characteristics of the enrolled patients are summarized in **Table 1**. There were no significant differences in age, gender, body mass index (BMI), American Society of Anesthesiologists (ASA) score, preoperative albumin concentration, preoperative hemoglobin concentration, primary tumor location, preoperative serum carcinoembryonic antigen (CEA) level between the two groups (**Table 1**). The interval from SEMS placement to operation was 10.0 days (IQR 7.25-13.75) in Group 1 and 91.50 days (IQR 50.75-122.50) in Group 2.

**Table 1:** Demographics and baseline characteristics of patients between Group 1 and Group 2.

	Group1 (n=104)	Group2 (n=44)	P value
Age (year, mean ± SD)	62.92 ± 13.12	58.41 ± 12.59	0.055
BMI, kg/m² (mean ± SD)	22.26 ± 4.22	21.22 ± 2.86	0.138
Gender, N%			0.305
Male	75 (72.1)	28 (63.6)	
Female	29 (27.9)	16 (36.4)	
Hemoglobin (g/L) median, (IQR)	115.00 (104.25-124.00)	107.0 (97.75-124.75)	0.746
Albumin (g/L) median, (IQR)	36.60 (32.08-39.50)	35.70 (32.18-38.78)	0.666
Tumor location, N%			0.098
Splenic flexure	17 (16.3)	2 (4.5)	
Descending	42 (40.4)	25 (56.8)	
Sigmoid	30 (28.8)	9 (20.5)	
Rectum	15 (14.4)	8 (18.2)	
Preoperative CEA, N%			0.427
Normal	38 (38.4)	20 (45.5)	
Elevated	61 (61.6)	24 (54.5)	
ASA class, N%			0.105
I	13 (12.5)	11 (25.0)	
II	79 (76.0)	31 (70.5)	
III	12 (11,5)	2 (4.5)	
pT status, N%			0.065
T2	0 (0)	2 (4.5)	
T3	53 (54.1)	27 (61.4)	
T4	45 (45.9)	15 (34.1)	
pN stage, N%			0.576
N0	44 (44.9)	20 (46.5)	
N1	35 (35.7)	12 (27.9)	
N2	19 (19.4)	11 (25.6)	

### Adverse events

There were 3 patients who developed stent-related adverse events. ESGE guidelines suggest an interval time of 2 weeks between SEMS placement and surgery, to balance stent-related adverse events (reduced by a short interval) and surgery-related adverse events (improved by a longer delay). In this study, stent-related adverse events occurred in 3 patients with an interval of ≤2 weeks between SEMS implantation and surgery, including perforation, occurred in 1 patient and stent migration occurred in 2 patients. However, no stent-related complications were observed in the interval of >2 weeks between SEMS implantation and surgery. In this study, it was not found that the stent-related adverse events were related to the longer time interval, which may be related to the small number of patients and the deviation of retrospective study. In a large prospective study, Saito et al. reported that most of the stent-related complications occurred within 7 days (14). Besides, surgery-related adverse events happened in 12 (14.8%) patients who underwent an interval of ≤2 weeks between SEMS implantation and surgery, and in 3 (13.0%) patients who underwent an interval of >2 weeks between SEMS implantation and surgery.

### Operation characteristics

Of the 148 patients, 70.27% of patients underwent surgery ≤4weeks of SEMS placement while 29.73% of patients underwent surgery >4 weeks of SEMS placement. Significant differences were observed between both groups for postoperative complications (Clavien-Dindo grading ≥II, 14.4% vs 2.3%, p=0.040), postoperative bowel function time (p<0.001), postoperative hospital stay (p=0.028) and total hospital stay (p=0.002). This study showed that compared with patients with SEMS placement interval ≤4weeks, patients who underwent neoadjuvant chemotherapy after SEMS placement (interval >4 weeks) had significantly shorter total hospital stay and postoperative bowel function recovery time; and the incidence of postoperative complications was significantly lower. There were no differences in intraoperative blood loss, surgical approach, number of lymph nodes examined, number of metastatic lymph nodes, vascular invasion, lymphatic invasion, operative time, and postoperative tumor markers between the two groups (**Table 2**).

**Table 2:** Early Surgical outcomes between Group 1 and Group 2.

	Group1* (n=104)	Group2* (n=44)	P value	Multivariate		
				Odds Ratio	95% Confidence Interval	P value
Number of harvested lymph nodes			0.722			
<12	7 (7.3)	2 (4.8)				
≥12	89 (92.7)	40 (95.2)				
Lymph nodes metastasis			0.652			
No	44 (45.8)	21 (50.0)				
Yes	52 (54.2)	21 (50.0)				
Perineural invasion			0.076			
No	46 (47.9)	27 (64.3)				
Yes	50 (52.1)	15 (35.7)				
Vascular invasion			0.288			
No	67 (69.8)	33 (78.6)				
Yes	29 (30.2)	9 (21.4)				
Surgical approach, N%			0.816			
Laparoscopic	89 (85.6)	37 (84.1)				
Open	15 (14.4)	7 (15.9)				
Postoperative complications (Clavien-Dindo ≥2), N%			**0.040**	0.071	0.006-0.847	**0.036**
No	89 (85.6)	43 (97.7)				
Yes	15 (14.4)	1 (2.3)				
ICU, N%			0.106			
No	96 (92.3)	44 (100.0)				
Yes	8 (7.7)	0 (0.0)				
Postoperative CEA, N%			0.683			
Normal	55 (75.3)	28 (71.8)				
Elevated	18 (24.7)	11 (28.2)				
Primary anastomosis	95 (91.3)	42 (95.5)				
stoma, N%			0.975			
No	73 (70.2)	31 (70.5)				
Yes	31 (29.8)	13 (29.5)				
Hartmann procedure, N%			0.507			
No	95 (91.3)	42 (95.5)				
Yes	9 (8.7)	2 (4.5)				
Postoperative bowel function (days), median, (IQR)	3.00 (3.00-5.00)	3.00 (2.00-3.00)	**<0.001**	0.527	0.359-0.774	**0.001**
Postoperative hospital stay (days), median, (IQR)	10.00 (7.00-13.00)	8.00 (7.00-10.00)	**0.028**	1.329	1.118-1.580	**0.001**
Total Hospital stay (days), median, (IQR)	22.00 (18.00-26.00)	15.00 (12.25-17.75)	**<0.001**	0.762	0.680-0.854	**<0.001**
Surgery time (min), median, (IQR)	196.50 (166.25-261.15)	210.00 (178.50-270.00)	0.845			
Intraoperative blood loss (ml), median, (IQR)	50.00 (50.00-125.00)	50.00 (50.00-100.00)	0.727			

Bold values indicates p-values<0.05 and was considered statistically significant.

### Postoperative complication

Fewer total postoperative complications occurred in Group 2 compared to Group 1 (2.3% vs 14.4%, p=0.040). In the Group 1, the most common complication was anastomotic leakage (4 cases, 26.7%), followed by lung infection (3 cases, 20.0%), abdominal wound infection (2 cases, 13.3%), postoperative intestinal obstruction (2 case, 13,3%), abdominal infection (1 cases, 6.6%), organ failure (1 case, 6.6%), surgical site infection (1 case, 6.6%), and urinary tract infection (1 case, 6.6%), seen in **Table 3**.

**Table 3:** Details of all complications after surgery between two groups.

Postoperative complications	Group1 (n=15)	Group2 (n=1)
Anastomotic leakage	4	0
Abdominal infection	1	0
Organ failure	1	0
Iung infection	3	0
Urinary tract infection	1	0
Abdominal wound infection	2	0
Postoperative intestinal obstruction	2	1
Surgical site infection	1	0

To exclude the influence of confounding factors on the results, multivariate logistic regression analysis was performed for variables with p value <0.05 in univariate analysis. The results of multivariate regression analysis showed that the results of the interval between stent implantation and operation were independent of each other, and the influence of confounding factors on the results could be excluded.

## Discussion

This study found that a time interval >4weeks from SEMS placement to elective surgery not only accelerated postoperative recovery, but also reduced postoperative complications. Treatment decision-making for LMCO remains a challenging problem, often with emergency surgery, resulting in multistage surgery and the creation of a stoma (15). SEMS can be used as a surgical bridge for elective surgery, and endoscopic decompression using SEMS can transform emergency surgery into one-stage elective surgery (16). SEMS placement as a BTS offers many theoretical advantages not only to correct problems such as fluid-electrolyte disturbances and cardiorespiratory function, but also to improve nutritional status. The main purpose of BTS after SEMS placement may be to perform tumor surgery in a more stable or improved physical condition in patients with obstructive colon cancer (17, 18). To achieve this, the optimal time interval between SEMS placement and elective surgery must first be determined. However, information on the appropriate time interval between SEMS placement and surgery and its impact is lacking. The ESGE in 2014 recommended a bridging interval of 5-10 days for patients with resectable obstructive left-sided colorectal cancer because of the potential for stent-related complications of more than one week, which may compromise surgery result. In 2020, the latest ESGE guidelines recommend an interval of approximately 2 weeks, which states that the interval before surgery should be determined by optimization of nutritional status and adequate management of comorbidities. This is also based on low-quality evidence and weak recommendations. For patients with locally advanced colon cancer treated with neoadjuvant chemotherapy, only a minority of patients have undergone colonic stenting as a bridge to surgery, and separate data are not available. Therefore, ESGE cannot make recommendations for this because the strategy for neoadjuvant chemotherapy during the bridging interval from SEMS placement to elective surgery has not been established (10).

Appropriate prolongation of the interval between SEMS placement and elective surgery is necessary to improve the clinical status of patients and resolve intestinal obstruction (19). A retrospective study reported that a time interval >15 days between SEMS placement and elective surgery could reduce postoperative complications (20). Lee et al. reported that the rates of anastomotic leakage were significantly higher in patients who had <10 days interval between SEMS insertion and surgery (21). In our study, compared with patients with a ≤4 weeks interval, patients with a >4 weeks interval had lower rate of postoperative complications. Prolonging the interval between SEMS and elective surgeries allows intestinal patency and anti-infective therapy to better restore intestinal barrier function, which making it difficult for bacteria to pass through the intestinal barrier. Therefore, it can reduce the risk of perioperative infectious complications. Some studies showed that postoperative complications may affect patients’ long-term survival with colorectal cancer (22, 23). In our study, prolonging the interval between SEMS placement and elective surgery was associated with fewer postoperative complications. Postoperative complications have a negative impact on survival after resection in cancer patients. In addition, the improvement of intestinal wall edema and ischemia also favors the modification of destructive microbes, which would reduce the occurrence of anastomotic leakage. Several studies have elucidated pro-inflammatory cytokines such as tumor necrosis factor-α, interleukin-6, and interleukin-8, has been implicated in promoting the growth of cancer cells (24). In theory, a longer bridging interval should improve gut condition and reduce the risk of anastomotic leakage. In addition, longer periods may provide more opportunities for improvement in the patient’s general condition. However, the underlying mechanism is unclear, as this status is not associated with any clinicopathological parameters. A nationwide, population-based study showed that the time interval between SEMS placement and elective surgery is not associated with postoperative complications (25). Our study showed that stent-related adverse events occurred in 3 patients with an interval of ≤2 weeks between SEMS implantation and surgery. A prospective multicenter study showed that the majority of stent and surgical adverse events occurred within a 7 days interval. Adverse events such as perforation may result from proximal colon dilatation away from the stent site due to insufficient decompression of the colon (26).

Further studies are needed to confirm the relationship between interval and postoperative complications and to elucidate the underlying mechanisms. Marnix et al. analyzed 168 patients with LMCO showed that a BTS interval of >4 weeks after SEMS placement was suggested for better short-term outcomes which including postoperative complications, postoperative hospital stay, and 90-day mortality (27). A retrospective study showed a bridging interval of > 2 weeks between BTS SEMS placement and surgery for LMCO is favorable for short-term clinical outcomes. It can not only form a lower stoma rate but also have a higher rate of laparoscopic surgery (28). In general, a longer interval could compromise surgery by more local tumor infiltration and fibrosis. However, our study showed that there are no significant differences in duration of surgery and intraoperative blood loss between patients who underwent surgery ≤4 weeks and >4 weeks after SEMS placement.

SEMS placement achieves the purpose of temporary decompression, which would not only help to eliminate the adverse effects of edema after intestinal obstruction on radical surgical operations, but also would reduce the difficulty of elective surgery. SEMS placement also buys time for bowel preparation to improve bowel blood supply, adjust the nutritional status of patients, reduce surgical risks, reduce postoperative complications such as anastomotic leakage, and speed up postoperative recovery (7, 8, 29). In addition, SEMS implantation is a palliative treatment, which helps to reduce postoperative pain compared with enterostomy, to shorten the postoperative hospital stay and to promote the early recovery of patients. In this study, after SEMS placement, the interval between radical tumor resections was prolonged to gain sufficient time for the bowel wall edema to subside (30). Our research points out that compared with patients with a ≤4weeks interval, patients with a >4 weeks interval had shorter total hospital stay and faster bowel function recovery. In conclusion, our findings suggest that a longer interval (>4 weeks) between SEMS placement and surgery does not affect surgical difficulty, but reduces postoperative complications, shortens hospital stay, and accelerates recovery of bowel function.

Compared with SEMS placement in patients with LMCO, whether prolonging the SEMS placement interval for neoadjuvant affects elective surgery and postoperative perioperative recovery of patients is unclear, and few studies have reported this question.

Micrometastases may already appear around the tumor tissue, and it is impossible to determine whether these micrometastases are removed during local excision when colorectal cancer develops into intestinal obstruction (31). Therefore, surgical experts have reached a consensus to eliminate micrometastases by chemotherapy before surgery. It could reduce the volume of the primary tumor, thereby providing good resection conditions for subsequent surgical resection (32). The efficacy of neoadjuvant chemotherapy for patients with locally advanced cancer is definite, which creating an opportunity for patients with radical resection and better prognosis. Implantation of SEMS to relieve obstruction not only avoids emergency surgery, but also creates an opportunity for preoperative neoadjuvant chemotherapy. It changes emergency surgery into time-limited surgery, reduces tumor volume, effectively eradicates micrometastases, and reduces tumor staging. So that it could greatly improves surgical efficacy and increases the chance of complete resection (13, 33). Adequate bowel preparation prior to surgery reduces perioperative risks and allows patients to recover faster and better after surgery. Our study believes that for LMCO, prolonging the interval between SEMS placement and elective surgery, receiving neoadjuvant chemotherapy in the next time period, appropriately prolonging the waiting time for surgery, does not increase SEMS-related complications, and the adverse reactions of chemotherapy are controllable, which can reduce postoperative complications, increase surgical safety, and speed up patient recovery.

A limitation of this study is that it is prone to selection bias due to its retrospective design. Therefore, this study should be interpreted with caution, and larger prospective studies are needed to validate our findings.

## Conclusions

For LMCO, longer bridging interval between SEMS placement and elective surgery (> 4 weeks) significantly reduced postoperative complication rates and faster recovery time and total hospital stay.

## Data availability statement

The raw data supporting the conclusions of this article will be made available by the authors, without undue reservation.

## Ethics statement

The studies involving human participants were reviewed and approved by institutional ethics board of the Sixth Affiliated Hospital, Sun Yat-sen University (NO. 2022ZSLYEC-429). Written informed consent for participation was not required for this study in accordance with the national legislation and the institutional requirements.

## Author contributions

All authors have read and approved the manuscript. Study conception and design: XG, JL. Administrative support: DL, QZ, JH. Acquisition of data: ML, TX. Analysis and interpretation of data: JD, MS, JZ. Manuscript writing: All authors. All authors contributed to the article and approved the submitted version.

## Funding

This work was supported by National Key Clinical Discipline, Natural Science Foundation of Tibet Autonomous Region, China (2031021016).

## Conflict of interest

The authors declare that the research was conducted in the absence of any commercial or financial relationships that could be construed as a potential conflict of interest.

## Publisher’s note

All claims expressed in this article are solely those of the authors and do not necessarily represent those of their affiliated organizations, or those of the publisher, the editors and the reviewers. Any product that may be evaluated in this article, or claim that may be made by its manufacturer, is not guaranteed or endorsed by the publisher.

## Abbreviations

LMCO, left-sided malignant colorectal obstruction; ICU, intensive care units; BTS, bridge to elective surgery; SEMS, self-expanding metallic stent; ESGE, European society for gastrointestinal endoscopy; CT, computed tomography; DSA, digital subtraction angiography.
